# Public health nurses’ experiences with work aimed at increasing adolescents’ health literacy: a qualitative study

**DOI:** 10.1186/s12912-025-03976-z

**Published:** 2025-10-28

**Authors:** Eden van der Schuur, Hilde Timenes Mikkelsen

**Affiliations:** 1 Department of Childhood and Education, Kristiansand Municipality, Kristiansand, Norway; 2 Department of Health and Nursing, Faculty of Health and Sport Sciences, University of Agder, Kristiansand, Norway

**Keywords:** Health literacy, Public health nurse, School health service, Adolescence, Public health

## Abstract

**Background:**

Health literacy is essential for maintaining good health by enabling individuals to make informed and beneficial health choices in their daily lives. Enhancing health literacy in adolescents is important, because it can empower them to become more engaged with and actively participate in their own health. More knowledge is needed on how public health nurses work to enhance health literacy among adolescents. Therefore, the objective of this study is to explore public health nurses’ experiences with work aimed at enhancing adolescents’ health literacy.

**Methods:**

The study was inspired by a phenomenological-hermeneutic approach. A qualitative research method was used, in which eight public health nurses from the school health service in the southern part of Norway were interviewed individually. The data were analyzed using Braun and Clarke’s thematic analysis approach.

**Results:**

Three main themes were identified: (1) Public health nurses use multiple approaches to enhance adolescents’ health literacy; (2) Health information during adolescence is influenced by social media; and (3) Parental involvement is crucial during adolescence.

**Conclusions:**

This study demonstrates the unique position of public health nurses in enhancing health literacy among adolescents and their parents. A core component of their work is focused on normalizing aspects of adolescents’ everyday lives, such as emotions and body image, which is vital considering adolescents’ exposure to unrealistic social media images and misleading health information found online. Furthermore, public health nurses play an important role in maintaining parental involvement, and they offer reliable, evidence-based guidance and support to adolescents and parents.

**Clinical trial number:**

Not applicable.

**Supplementary Information:**

The online version contains supplementary material available at 10.1186/s12912-025-03976-z.

## Background

Health literacy (HL) is essential for maintaining good health by enabling individuals to make conscious and beneficial health choices in their daily lives [1]. HL refers to an individual’s ability to access, understand, appraise, and use health information to make informed decisions regarding their own health [2]. Nutbeam suggested three levels of HL: (1) Functional HL involves basic reading and writing skills and knowledge about the body, which are necessary for understanding health information and following healthcare instructions; (2) Interactive or communicative HL involves the ability to obtain and comprehend health information and to engage in interactions with others to extend available information and make decisions; and (3) Critical HL requires skills in critical thinking and evaluation of health information, as well as engagement in health promotion activities and addressing societal health issues, contributing to informed decisions and public health advocacy [3].

HL is important for adolescents, who generally have good health and are considered a healthy group in the population [4], HL can empower adolescents to become more engaged with and actively participate in their own health [5]. Conversely, insufficient HL not only poses a risk to adolescents but also impacts society by leading to poorer health outcomes and increased costs [5, 6]. Hence, this life phase presents an opportunity to enhance HL and promote positive health behaviors, as research indicates that HL can positively influence adolescents’ health in the present and over time, extending into adulthood [5, 7].

Adolescence is the transitional phase between childhood and adulthood, representing a unique stage for development and growth [8, 9]. This phase is characterized by increasing independence, as adolescents begin to make more decisions of their own while gradually distancing themselves from their parents [8, 10]. In this process, adolescents are generally expected to take greater responsibility for their own health, which places higher demands on their ability to find and evaluate health information [6, 11]. Furthermore, adolescence is a crucial period in life when habits related to lifestyle and health are established, which may persist into adulthood [8, 9, 12]. Adolescents tend to be highly focused in the present moment, which can encourage full engagement with current experiences, but it may also limit their capacity to consider long-term consequences or make future-oriented decisions related to their health [13, 14]. Hence, adolescents still need parental and/or adult support until they mature further in their overall development [6, 12].

Adolescents are exposed to health information that varies in quality, often through social media, covering topics such as sexual and mental health, chronic and infectious diseases, exercise, and nutrition. This information is often conveyed by influencers and commercial and non-commercial actors who have diverse intentions and are subject to minimal regulations [15–17]. These parties may, for instance, portray unhealthy habits in a positive light, promote harmful body ideals and focus excessively on exercise and diet [16]. Within an increasingly digitalized society, the need for digital HL is increasing. This involves the ability to search for, find, understand, evaluate, and apply health information from digital sources [18]. Therefore, it is crucial for adolescents to be able to critically analyze this information and understand its purpose and intent [4].

Public health nurses (PHNs) play a crucial and central role in public health by empowering children, adolescents, and families with information and guidance that can enhance their confidence and independence in making health decisions [19, 20]. Their work centers on health promotion and disease prevention and can be carried out at the individual, group, or population level [19]. Health promotion primarily focuses on strengthening overall health and is based on positive resources and factors that contribute to health maintenance and quality of life. Preventative work in the health field is based on scientific knowledge about the causes and factors that lead to illness and how these factors can be counteracted [7]. In working with adolescents, PHNs can promote health and work preventatively by enhancing their HL through education, counseling, and guidance [4]. For example, PHNs can play a vital role in guiding adolescents through the health information they encounter on social media [4].

In Norway, PHNs meet nearly all the adolescents with whom they work through the school health service, making this service unique in its ability to reach the adolescents at school [21]. A sustainable school health service should prioritize group-level health promotion and prevention to meet adolescents’ general need for enhanced HL while providing individual support for those in need [4]. Adolescents can benefit from professional guidance to correct misconceptions around health and from the support they receive regarding health-related issues [22, 23]. Interdisciplinary collaboration between professionals working with adolescents is considered crucial for effective preventive and health-promoting efforts, including active involvement in school programs related to HL [24–26]. Therefore, the school health service should actively participate in delivering health education to various student groups and classes. In this respect, Yu et al. [27] recommended adopting participatory learning, as simply delivering information in a one-sided and passive manner is unlikely to result in behavioral change, while engaging adolescents in the learning process is vital for user involvement and supporting health-promoting efforts [22, 27]. However, understanding the cognitive development of adolescents is crucial for effectively addressing health education. For instance, a Norwegian study among adolescents in lower secondary schools suggested that younger adolescents may not be cognitively ready to absorb and understand specific knowledge about mental illnesses and their symptoms. The study showed that tenth graders were better at recognizing symptoms than eighth graders [24].

To date, there is limited research and knowledge about strategies to strengthen or improve adolescents HL. More knowledge is needed, as this will allow for the development of more targeted and effective HL interventions tailored to adolescents’ unique developmental needs, thus ensuring that efforts are well-directed and impactful. Furthermore, a more comprehensive understanding of how PHNs work to enhance adolescents’ HL is needed. Thus, the objective of this study is to explore PHNs’ experiences with work aimed at enhancing adolescents’ HL.

## Methods

### Study design

Inspired by a phenomenological-hermeneutic approach, this study employed a qualitative approach through individual semi-structured interviews with PHNs to explore their experiences with work aimed at enhancing adolescents’ HL. This approach is suitable for exploring lived experiences, as it provides insight into how PHNs experience and interpret their work, emphasizing both the description of phenomena as experienced and the interpretation of its meaning within context [28, 29]. The approach was chosen to access the PHNs’ subjective life world and to deepen the understanding of their experiences.

### Data collection

The participants were purposively sampled, which means they were selected for their ability to provide rich and relevant information about the phenomenon under study [29], and they were recruited from lower secondary schools in the southern part of Norway. The first author, who was responsible for recruiting the PHNs, contacted their leaders in school health services in several municipalities, providing information about the study and obtaining permission to contact the PHNs directly. The PHNs were subsequently sent an open invitation to participate by email. Eight PHNs signed up to participate in the study.

Conducting research within one’s own field offers advantages due to the interviewer’s background, such as an enhanced understanding of the PHN profession and the school health services. However, this familiarity can make the researcher’s perspective too dominant. Thus, efforts were made to set aside preconceptions, enabling PHNs to share their experiences openly. Regular discussions between the first and second author helped analyze the influence of their background on the study’s decisions and methods.

An interview guide was developed for this study to obtain the most comprehensive insight into how PHNs in lower secondary schools engaged with HL and how they experienced this work (Supplementary file 1). The interview questions were formulated based on prior research and theories related to the topic, aiming to achieve a deeper understanding. One of the first questions in the interview guide was, “What are your thoughts on the health literacy concept’’, which had the intention of eliciting their personal reflections and thoughts.

The interviews were conducted either in-person or digitally, lasting 30–40 minutes, and were conducted and transcribed verbatim by the first author between May and June 2024. The in-person interviews took place at the participants’ workplaces, and the digital interviews were conducted using the digital platform Zoom. The participants were encouraged to openly share their experiences, and subsequent questions were tailored to their responses. The interviewer used “mirroring’’ and follow-up inquiries to ensure alignment between her interpretations and the participants’ understanding, especially when the descriptions were unclear or implicit.

### Analysis

The interviews were analyzed following the six steps of reflexive thematic analysis developed by Braun and Clarke [28]. The first step was to become familiar with the data by repeatedly listening to recordings and noting down emerging ideas and themes during transcription. In step two, meaning units were identified and systematically coded. These codes were divided into themes with subthemes. In step three, the themes were reviewed in relation to the study objective, and three main themes were identified. The codes were based on shared ideas or meanings, and the themes were actively constructed by the researchers. Step four involved a critical examination of the identified themes to ensure that the preliminary theme titles were logical and descriptive for the entire dataset. This step also included evaluating whether themes should be merged, split, or discarded [28]. In step five, the themes and subthemes were refined and defined to reflect their core content. The names of the themes were intended to be informative, capture the essence of the theme, and engage the reader [28]. The final step was the presentation of results, using representative quotations to illustrate each theme. The analysis process was data-driven and mainly done by the first author, with reflexive discussion and guidance from the second author to ensure a shared understanding of the themes. NVivo software [30] was used for data organization and analysis. The researchers collaboratively selected quotations that best reflected the identified themes. Reflexivity was consistently integrated, with researchers reflecting on their assumptions and interpretations to maintain a participant-centered analysis while recognizing their influence on the findings.

### Ethical considerations

The project was ethically approved by the Norwegian Agency for Shared Services in Education and Research (Ref: 997749) and the Faculty of Health and Sport Sciences at the University of Agder. Participation in this study was voluntary for all participants, and their written consent was obtained in advance. During transcription, the participants were anonymized by identifying them only by numbers. Thus, the quotations presented in the results are linked to the participants’ numbers. All collected data material was securely stored in a password-protected user area on the university server.

## Results

Eight PHNs aged 34–60 years who worked in lower secondary schools participated in the study. All were female and had obtained further education in public health nursing. Their years of seniority as PHNs ranged from 6 to 28 years. Four participants worked as PHNs in a large city, while the others worked in the district (Table 1). Three main themes were identified: (1) Public health nurses use multiple approaches to enhance adolescents’ health literacy; (2) Health information during adolescence is influenced by social media; and (3) Parental involvement is crucial during adolescence. The participating PHNs are cited by numbers (PHN 1, PHN 2, and so on).

**Table 1:** Participant characteristics

Participant	PHN seniority (years)	Further education	City/district	
PHN 1	12	Yes	City	
PHN 2	28	Yes	City	
PHN 3	6	Yes	District	
PHN 4	18	Yes	District	
PHN 5	3	Yes	District	
PHN 6	18	Yes	City	
PHN 7	16	Yes	District	
PHN 8	19	Yes	City	

### Public health nurses use multiple approaches to enhance adolescents’ health literacy

None of the participants felt that they were well acquainted with the term HL, as it was not a term they used or talked about in their daily work. However, they all said that they unconsciously worked with HL on a daily basis. The participants viewed HL as encompassing knowledge about health and acquiring knowledge about what is good for our health, both physically and mentally. Hence, they considered HL to be an important and central focus of their work because adolescents need to receive correct information and essential tools to make informed decisions about their health. Regarding adolescents’ HL, one participant stated:

*When I think about it, HL is about what adolescents know, and what kind of knowledge adolescents have about what is good for their health and what is harmful, what can affect their health in a positive and negative way. (PHN 8)*

The participants described working to increase HL among adolescents and their parents in many ways and on individual and group levels. Most of the participants believed that the main goal of working as a PHN was to promote health and prevent disease, which involved enhancing the HL of adolescents and their parents.

All the participants believed that they had a unique position in the school health service. They mentioned that both adolescents and teachers said they found it reassuring to have a PHN present at school. The participants strongly believed in being accessible to the adolescents and felt that many adolescents needed a relationship with the PHN and a place where they could openly discuss their concerns.

An important aspect of their work that all the participants mentioned is the requirement for PHNs in Norway to conduct a health conversation with all adolescents in the eighth grade. These conversations were used to convey knowledge to adolescents that could help them make good health choices, and they were considered a unique way to enhance adolescents’ HL on an individual level. According to the participants, this allowed them to obtain an indication of eighth-grade adolescents’ HL level, and they were able to adapt the information topics in the conversation to each individual adolescent and their needs.

All the participants stated that teaching is part of the PHN’s responsibilities and described it as a method of working with adolescents at the group level together with diverse professional groups such as teachers, physiotherapists and doctors. They regarded this as an effective method to enhance adolescents’ HL. The participants all said that their experiences with teaching were positive and that they felt that their voices were heard by the adolescents. They expressed that the best approach to teaching involves engaging adolescents with topics that are pertinent to them. This could be done by presenting various health-related statements and encouraging them to think critically. Additionally, they emphasized the importance of providing explanations and engage the adolescents in discussions rather than simply lecturing. Some participants mentioned that a challenge in teaching adolescents was that their receptiveness depended on their stage of development, leading to significant differences in what they found interesting or relevant. One participant explained it this way:

*But the challenge is that they are so different… A thirteen-year-old is not just a thirteen-year-old… they are all so different, with varied experiences and at different stages in life. Reaching everyone with a single, shared lesson can be difficult. (PHN1)*

### Health information during adolescence is influenced by social media

The participants stated that they felt they were competing with social media in their efforts to provide health information to adolescents. One participant mentioned that PHNs are up against very powerful forces in social media and emphasized that it is more important than ever that adolescents turn to healthcare professionals to ensure they receive accurate health information.

*We are also competing with… of course, information from parents, but that’s not so bad. The real challenge is social media, influencers, and the things they come up with*–*many of which are completely wrong. (PHN 2)*

All the participants mentioned social media platforms, such as TikTok, Instagram, and Snapchat, as adolescents’ primary sources of health information. They emphasized that social media exposes adolescents to content about weight, food, and exercise, that is often problematic because its quality is frequently not assured. The participants believed that influencers should be more aware of their impact, as today’s adolescents are much more affected by them than ever before. Furthermore, all the participants noted that adolescents frequently accept health information found online or on social media as truths. Three participants conveyed that source criticism poses a challenge for adolescents, as they are not equally adept at distinguishing between reliable and unreliable information.

The participants described an increasing need to normalize the challenges adolescents face in life and suggested that these could be linked to the health-related information adolescents encounter on social media. Two participants pointed out that, particularly within the mental health field, it has become common for adolescents to diagnose health issues and label them accordingly. Many adolescents lack a full understanding of terms such as “anxiety” and “depression” and frequently misuse these when describing how they feel. One participant shared that she had attempted to clarify these terms with eighth-grade students, and it was apparent that many were not fully aware of the difference between being depressed and feeling sad or being afraid of something and having anxiety. One participant described working with normalization this way:

*So, it’s a really important job we do—to normalize the concept. Or not the concept, but everyday life and living. It’s normal to have bad days, to feel down, grumpy, or sad without… without being mentally ill because of it. That’s an important part of our job. (PHN 3)*

### Parental involvement is crucial during adolescence

The participants described adolescence as a turbulent period in which adolescents are trying to find their place of belonging and gain greater independence from their parents. However, several participants emphasized that parental support and involvement remain essential throughout this stage. It was noted that parents play a crucial role in the support networks of adolescents. Hence, it was considered vital to make parents aware that adolescents are not yet mature enough to handle everything independently and that they still need their parents’ support, even though they may not ask for it. Several participants had the impression that most adolescents feel more secure when their parents are engaged and informed about their health.

*But most adolescents are kind of like: yeah, but we need mom… it feels safe to have mom and dad with us if we’re going to the doctor or things like that. (PHN 2)*

One participant explained that involving parents is always done in collaboration with adolescents. Although confidentiality sometimes prevents this, consistent efforts are made to keep the parents involved. According to several participants, the PHN can act as an important mediator in preserving and enhancing the relationship between adolescents and their parents.

All the participants stated that they primarily communicated with parents through school-organized parental meetings, and they reported having had numerous positive experiences in these meetings. However, they also expressed that their time at parental meetings was limited, often only 10–15 min. All the participants conveyed a wish to be allocated more time during parental meetings, as they felt that parents often forget that PHNs exist and their potential as resources. One participant believed that parents felt supported when they found out that PHNs conveyed the same message to adolescents that the parents themselves had been trying to communicate:

*But parents feel supported. We communicate a lot of the same things they do, and that can be reassuring for the adolescents to hear—like, okay, maybe what mom or dad is saying isn’t so wrong after all. (PHN 6)*

To enable parents to make informed health-related decisions for their adolescents, the PHNs emphasized the need for both knowledge and competence. One participant noted that many parents seem to lack the necessary knowledge to determine what is best for their adolescents and thus frequently adhere to what every other parent is doing without questioning these trends or practices. The participants emphasized the need for parents to possess critical thinking skills to make informed, evidence-based decisions. This was considered something the PHNs could help them with. However, the participants stressed the importance of adopting a humble approach when interacting with parents. One participant described it this way:

*I also feel when you’re talking to adolescents and their parents, they can be a bit defensive… like, ‘You’re not going to come here and tell me what to do with my child’. So, it’s really important to present things in a way that makes them feel they have autonomy and can make their own choices. (PHN 5)*

## Discussion

This study aimed to provide a deeper understanding of PHNs’ experiences with work aimed at enhancing adolescents’ HL. Three main themes were identified: (1) Public health nurses use multiple approaches to enhance adolescents’ health literacy; (2) Health information during adolescence is influenced by social media; and (3) Parental involvement is crucial during adolescence.

### The multifaced role of public health nurses in promoting health literacy

Our study identified that PHNs had limited familiarity with the HL term. This could be explained by the fact that this term was formally introduced in Norway in 2019, after their PHN training. Despite this, all the PHNs reported that they unconsciously engaged with HL-related activities daily, utilizing various strategies to enhance adolescents’ HL at the individual and group levels. At the individual level, PHNs supported adolescents through health conversations in eighth grade and maintained an “open door” policy, providing a safe space for conversations. Previous studies have emphasized that the ability to listen to and give time to adolescents to express themselves is fundamental when working on their mental health [31, 32]. In promoting their mental health, studies have highlighted the importance of affirming and focusing on adolescents’ self-esteem and taking an optimistic perspective of their situation [31, 33]. Our findings indicate that individual health conversations, such as those in the eighth grade, can improve adolescents’ functional HL, which, according to Nutbeam [3], is essential for understanding health information and following health advice.

The results showed that educational interventions conducted within classroom settings and during parent meetings functioned as avenues for promoting HL at the group level. Education emerged as a key method for enhancing HL, with PHNs promoting health awareness through evidence-based information. This supports sustainable school health services [4]. Research by Oliveira Costa et al. [31, 34] showed that PHN-led classroom interventions improved adolescents’ mental health knowledge and mental HL, encouraging active participation in their mental health management. These interventions foster interactive HL through two-way communication and encouraging active engagement with health topics. Additionally, it supports critical HL by promoting dialogue. This approach empowers adolescents to question, reflect, and make informed health decisions as they become more independent. According to Haugland and Grimsmo [21], PHNs can play a key role in promoting HL, as they are present and accessible in the school environment and can reach all adolescents.

The PHNs emphasized the importance of engaging adolescents with pertinent topics, as this can enhance their active participation, which in turn improves their learning and helps them make informed health decisions. This perspective aligns with research by Dahl [22] and Yu et al. [27], which highlighted the role of participatory approaches in reinforcing HL in adolescents and encouraging desirable behavioral change. Furthermore, McAllister et al. [31, 35] underscored that health promotion efforts should prioritize dialogue and student-centered content rather than relying on one-way information of health-related content. Recognizing adolescents as experts in their own lives is seen as essential to ensuring their meaningful engagement. However, our study indicates that PHNs may encounter challenges during educational interventions due to adolescents’ diverse developmental stages and interests, supporting theories centered on adolescents’ cognitive development and the imperative for age-appropriate educational strategies [8, 10]. Consistent with this, Skre et al. [24] found that tenth-grade students were cognitively more mature and better able to process health information than eighth graders. This indicates the need for PHNs to be more involved in educational interventions in tenth grade, when adolescents are cognitively more mature.

All the PHNs in this study reported collaborating with different professional groups such as teachers, physiotherapists and doctors in the context of teaching HL-related topics. Examples included co-planning lessons and co-teaching health-related topics such as mental health, sexual health and nutrition. PHNs also joined school-based support groups with special education staff, social workers, and teachers to address individual adolescence’s needs. This aligns with previous research advocating interprofessional collaboration in school-based HL programs [24, 26]. Promoting health in the school context is considered more effective when school staff, parents, adolescents, and healthcare professionals are recognized as equally important collaborators working together [31, 36]. According to Hughes and Maiden [26], cooperation between schools and PHNs can help prepare adolescents to become their own health advocates as they begin to make independent health decisions. This emphasizes the importance of integrating diverse professionals into health-related educational interventions.

### Addressing social media’s influence on adolescents’ health literacy

The PHNs in this study noted that social media significantly influences the health information adolescents acquire, often leading them to accept social media content as factual without critical evaluation. This observation aligns with research by Taba et al. [17], showing that adolescents use platforms such as YouTube and TikTok to find information on lifestyle and health-related topics. Such trends indicate a need to strengthen adolescents’ digital, communicative, and critical HL, which according to Nutbeam [3] includes the ability to seek, assess, and evaluate health information. Distinguishing reliable from unreliable health information is crucial for informed decision-making and maintaining good health [2]. Strengthening these skills can empower adolescents to navigate the complex digital health landscape. PHNs can support this through practical interventions such as workshops and educational materials aimed at improving source evaluation skills, addressing Nutbeam’s critical HL level. Through health conversations and educational initiatives, PHNs can also foster interactive HL, encouraging dialogue and self-reflection.

Our results showed that normalizing adolescents’ body images, daily lives, and general life challenges was central to PHNs’ work. According to Øverland [37], the increasing need for normalization may be associated with the unregulated health information adolescents receive through social media. Social media contributes to body image pressure by enabling and encouraging individuals to edit and manipulate their appearance [37]. It also presents challenges related to influencers and people who may promote unhealthy attitudes toward exercise and diet [17]. This aligns with Glavin and Kvarme [23], who argued that adolescents need professional adult guidance on various health and well-being issues. Our results indicate that PHNs can be a valuable resource in providing such guidance.

The participating PHNs expressed concern about the misuse of terms like “anxiety” and “depression”, leading to risks of overdiagnosis and emotional unawareness, which seemed influenced by external health information. Myhre [13] noted that emotional challenges are common during adolescence, but they should not be overly pathologized, and it is essential to recognize adolescents as a healthy demographic. According to Holmen and Bjørnsen [4], knowledge about adolescent brain development and normal life variations can help raise awareness of what mental health entails and what behavior is typical during adolescence. These findings underscore the importance of proper guidance from PHNs and other professionals to help clarify normal emotions and thoughts during adolescence. PHNs should focus on normalizing everyday challenges and enhance HL through knowledge and attitude strengthening [2].

### Parental involvement is important to enhance adolescents’ health literacy

The PHNs in this study emphasized that parental involvement is both important and necessary throughout adolescence. According to Anttila et al. [31, 38], parents have a significant influence on their adolescents’ development. During this stage, parents and other adults should continue to provide support and security to help adolescents consider the long-term consequences when making future-oriented health decisions [13, 14]. However, our findings indicate that many parents mistakenly believe that they lose their influence once their adolescent enters lower secondary school. Hence, this study suggests that during this period, PHNs should raise parental awareness about the importance of remaining actively involved in their adolescents’ lives. This is particularly important for parents to consider if they wish to influence their adolescents before the age of 16, when society expects them to take responsibility for their own health [2].

Adolescents’ HL is influenced by their parents’ HL, and research indicates that parents with high HL are better at teaching essential health-related skills and providing resources to further develop these skills [7]. PHNs can empower parents to stay engaged as their children take on more health responsibilities. While PHNs in this study primarily interacted with parents during school-organized parental meetings, limited meeting time often posed challenges. Nevertheless, these meetings offered valuable opportunities to reach and engage with parents at a group level. Our study suggests allocating sufficient time and defining PHNs’ role during these meetings to maximize their impact. PHNs should also remain visible to both adolescents and parents. However, it’s important to consider structural and social barriers to parental engagement, such as time limitations, educational disparities affecting health information comprehension, and health inequalities limiting access to resources. Addressing these barriers is essential for creating an environment where PHNs and parents can collaboratively support adolescents’ well-being.

When PHNs encourage parental involvement, they may indirectly support adolescents’ functional HL, especially when parents are equipped to explain basic health concepts and guide everyday health decisions. Through school meetings and collaborative dialogue, PHNs may also foster interactive HL, by creating opportunities for parents to engage in meaningful conversations about health with both professionals and their adolescents. Furthermore, by helping parents critically reflect on health information, societal expectations, and digital influences, PHNs contribute to the development of critical HL.

According to Tveiten and Severinsson [20], by providing research-based guidance, PHNs can increase parents’ confidence in making informed health decisions for their adolescents. When PHNs and parents convey the same messages to adolescents, it can help strengthen the credibility of parental guidance, making adolescents more likely to take their parents’ advice seriously. In this way, parents and PHNs can collaborate in promoting for adolescents’ well-being.

### Strengths and limitations

The strengths of the study include reciprocal reflection among researchers on the analysis and findings, which enhances the reliability and trustworthiness of the insights obtained [29]. Participants’ quotations were presented to ensure transparency. The sample consisted of PHNs with diverse variations in age, seniority, and work location, thereby enriching the range of perspectives. However, the study was limited by a lack of gender diversity, as only female informants were included. This primarily mirrors the gender distribution within the PHN profession in Norway, where the vast majority are women, and thus does not imply a selective recruitment process. Additionally, the findings are solely based on PHNs’ experiences, which might differ from those of parents and adolescents. The interview format (in-person vs. digital) did not have any impact on the duration of the interviews. However, while digital interviews facilitated the study’s logistics, their less personal nature and the lack of non-verbal cues may have affected the spontaneity and depth of communication compared to the more engaging in-person interviews. These factors should be considered when interpreting the results. Despite these limitations, the study’s insights into strategies for improving adolescents’ HL may be applicable in similar contexts, informing broader approaches to providing adolescent health services.

### Implications for practice

The findings highlight the importance of increasing HL among adolescents, and the study offers strategies for PHNs in school health services to achieve this goal. We recommend that PHNs take a more active role in enhancing adolescents’ HL and prioritize this work within their daily practice, including providing extended information and support during parental meetings to enhance parental HL. Furthermore, we recommend that PHNs take a more active role in educational health interventions in the tenth grade, when adolescents are cognitively more mature.

Further research from the PHN, adolescent, and parent perspectives is needed. Adolescents should become a clearer target group in HL research, as existing studies indicate that research on adolescents’ HL remains limited compared to adults. Additional investigations of HL among adolescents could provide valuable insights into interventions and methods to maintain or improve their HL.

## Conclusions

This study demonstrates PHNs’ unique position in enhancing HL among adolescents and their parents. A core component of their work is focused on normalizing aspects of adolescents’ everyday lives, such as emotions and body image, which is vital considering their exposure to unrealistic social media images and misleading health information found online. Furthermore, PHNs play a crucial role in maintaining parental involvement by providing reliable, evidence-based guidance and support to adolescents and parents. The study underscores the importance of systematically integrating HL into school health services to equip adolescents with vital skills for navigating health information and making informed health decisions, which will benefit them into adulthood. Using Nutbeam’s three levels of HL (functional, interactive, and critical) as a framework, the study provides suggestions on how PHNs’ interventions can support each of these levels among adolescents and their parents.

## Supplementary Information

Below is the link to the electronic supplementary material.

Supplementary Material 1

## Data Availability

No datasets were generated or analysed during the current study.

## Abbreviations

HL: Health literacy
PHN: Public health nurse
