# Supplementary material for: Public health nurses’ experiences with work aimed at increasing adolescents’ health literacy: a qualitative study
Source: BMC Nurs. 2025 Oct 28;24:1333. doi: 10.1186/s12912-025-03976-z (PMC12560339; doi:10.1186/s12912-025-03976-z)
Supplement: Supplementary file 1 — Supplementary Material 1 [file 12912_2025_3976_MOESM1_ESM.docx]

**Interview Guide**
*Public health nurse's experience with work aimed at*

*increasing adolescents' health literacy*

**1. Introduction**

- Introduction of the researcher.
- Presentation of the research project and the purpose/objective of the project

**2. Health Literacy**

- What are your thoughts on the health literacy concept? (Are you well familiar with the term?)
- What experience do you have working with health literacy as a public health nurse? (Positive and negative experiences related to health literacy work)
- Do you actively incorporate this topic into your work in the school health service? What opportunities exist at your workplace to promote adolescents’ health literacy?

**3. Collaboration between Public Health Nurse, School, and Parents**

- Do you collaborate with teachers/other school staff on this topic? (Please elaborate on your experiences with this collaboration)
- Do you have experience from schools you work at/have worked at which are addressing this topic? If yes: How does/did the school address this topic?
- Do you have any experience from collaborating with parents on topics related to health literacy?
- Do you know if health literacy has been addressed at parent meetings at your school?

**4. Finding Health Information / Legal Health Autonomy**

- Where do you get the impression that adolescents obtain health information?
- Do you have experience with adolescents asking you about information they found on social media?
- Are adolescents at your school informed about becoming legally responsible for their own health decisions at age 16, and what this entail? (Why/why not?)

**5. Conclusion**

- Do you have any further thoughts or experiences related to this topic that you would like to share?
- How did you experience the interview?
